# Pseudocholinesterase Deficiency in a Patient Undergoing Electroconvulsive Therapy: A Case Report

**DOI:** 10.7759/cureus.98333

**Published:** 2025-12-02

**Authors:** António C Ladeira, João Laranjeira, Luciana Guariento, Muriel Lérias-Cambeiro, Paula Victor

**Affiliations:** 1 Anaesthesiology, Unidade Local de Saúde Santa Maria, Lisbon, PRT

**Keywords:** awareness, delayed emergence, electroconvulsive therapy, neuromuscular monitoring, pseudocholinesterase deficiency, rocuronium, succinylcholine, sugammadex

## Abstract

Pseudocholinesterase deficiency (PD) is a genetic or acquired condition that impairs the metabolism of succinylcholine, leading to unpredictable and potentially prolonged paralysis. Its recognition during electroconvulsive therapy (ECT) is particularly difficult because postictal physiology, psychotropic polypharmacy, and the frequent absence of quantitative neuromuscular monitoring may make residual paralysis difficult to detect. We report a 34-year-old woman with pharmacotherapy-resistant schizophrenia who developed consistently prolonged paralysis after succinylcholine during ECT. In the first session, anaesthesia with propofol (80 mg) and succinylcholine (60 mg) produced an adequate seizure, but recovery of spontaneous ventilation and motor function was delayed by approximately 24 minutes, with only mild tachycardia and hypertension; this was initially attributed to chronic psychotropic treatment. The same pattern recurred during the second session despite adequate hypnotic depth on bispectral index (BIS) monitoring and unreliable neuromuscular monitoring data. Laboratory testing subsequently revealed markedly reduced plasma pseudocholinesterase activity (3745 U/L), confirming PD. Succinylcholine was then replaced with rocuronium (40 mg), and reversal was performed with high-dose sugammadex (960 mg), resulting in immediate and uneventful recovery in all remaining sessions under continuous quantitative neuromuscular monitoring. This case illustrates that PD may go unrecognised in ECT when quantitative neuromuscular monitoring is unavailable or unreliable and reinforces the need for routine monitoring to detect atypical recovery patterns and reduce the risk of unrecognised awareness during anaesthesia.

## Introduction

Electroconvulsive therapy (ECT) is a well-established treatment for severe psychiatric disorders resistant to pharmacotherapy, such as major depression and schizophrenia [1-3]. The procedure requires general anaesthesia and the administration of a neuromuscular blocking agent (NMBA) to minimise the risk of musculoskeletal injury during the seizure.

Succinylcholine is the most commonly used NMBA in ECT owing to its rapid onset and brief duration of action [1,3]. Its metabolism depends on plasma pseudocholinesterase (butyrylcholinesterase) activity, and deficiency of this enzyme, whether genetic or acquired, can result in prolonged neuromuscular block [4,5].

Pseudocholinesterase deficiency (PD) may be hereditary, following an autosomal recessive pattern, or acquired due to conditions such as liver disease, malnutrition, pregnancy, malignancy, or exposure to drugs including monoamine oxidase (MAO) inhibitors and corticosteroids [6,7]. The prevalence is estimated at approximately one in 3,200 for homozygotes and up to one in 25 for heterozygotes [4,6].

Recognising PD during ECT can be particularly challenging. The procedure is performed rapidly, often outside the operating theatre, and neuromuscular monitoring is not always performed when succinylcholine is used, which may make it more difficult to detect residual neuromuscular block or recognise atypical recovery patterns. The postictal neurological state and concurrent psychotropic medication may further complicate interpretation. Therefore, objective neuromuscular assessment is strongly recommended to support early recognition of prolonged paralysis [8,9]. This case is presented to illustrate the practical challenges of identifying PD during ECT and to reinforce the importance of quantitative neuromuscular monitoring in ensuring timely recognition and safe anaesthetic management.

## Case presentation

In July 2022, a 34-year-old woman (height 162 cm, weight 60 kg, American Society of Anesthesiologists (ASA) physical status II) with pharmacotherapy-resistant schizophrenia was scheduled for 12 ECT sessions. Her chronic medications included clozapine 450 mg/day, paliperidone 150 mg monthly depot, trazodone 150 mg/day, and mexazolam 1 mg twice daily. She had no prior exposure to general anaesthesia, no history of anaesthetic complications, no family history of adverse reactions, and no known allergies.

During the first ECT session, standard ASA monitoring was applied (ECG, pulse oximetry, and non-invasive blood pressure). Anaesthesia was induced with propofol (80 mg) and succinylcholine (60 mg). The seizure lasted 22 seconds (motor) and 29 seconds (electrographic). Following the seizure, spontaneous ventilation and motor activity were absent for approximately 24 minutes. During this period, the patient remained mildly tachycardic and hypertensive. Manual bag-mask ventilation was maintained throughout, and after recovery, a Brice Interview revealed no recall of intra-procedural events.

Given the patient’s chronic psychotropic and antiepileptic regimen, the prolonged emergence was initially attributed to the sedative and pharmacokinetic effects of these medications on anaesthetic recovery. No laboratory investigations were performed at this stage, as the episode was interpreted as an exaggerated pharmacological response.

At the second session, the same anaesthetic technique was used with propofol (80 mg) and succinylcholine (60 mg). Again, recovery of spontaneous ventilation was markedly delayed. BIS monitoring indicated adequate hypnotic depth (55-65), while the peripheral nerve stimulator, used to deliver single-twitch stimulation, produced inconsistent and unreliable responses, preventing confirmation of residual blockade. The reproducibility of the clinical pattern raised suspicion of PD. Laboratory testing subsequently demonstrated a plasma pseudocholinesterase activity of 3745 U/L (reference range 5320-12920 U/L). Measurement of the dibucaine number was not available, but the clinical presentation, combined with markedly reduced enzyme activity, was sufficient to establish the diagnosis.

**Figure 1:**
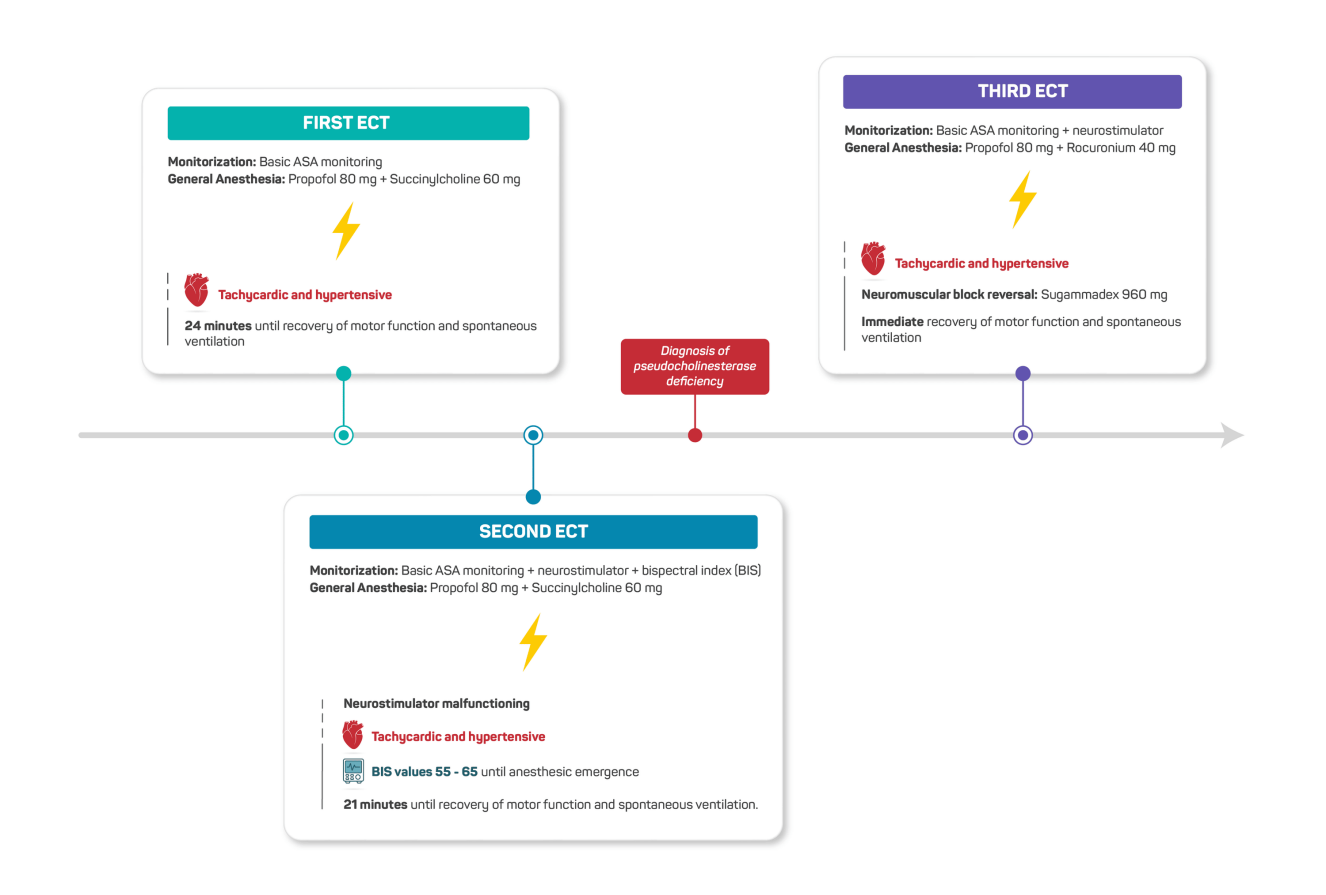
Timeline of the first three ECT sessions Timeline of the first three ECT sessions, highlighting recurrent prolonged recovery after succinylcholine administration, malfunctioning neuromuscular monitoring, and subsequent diagnosis of pseudocholinesterase deficiency. Switching to rocuronium with sugammadex resulted in immediate recovery. ASA, American Society of Anesthesiologists; BIS, bispectral index; ECT, electroconvulsive therapy

For all subsequent ECT sessions, succinylcholine was replaced with rocuronium (0.6 mg/kg, approximately 40 mg), and immediate reversal was achieved with high-dose sugammadex (16 mg/kg, approximately 960 mg) administered after the seizure to ensure rapid and predictable recovery outside the operating theatre. Quantitative train-of-four (TOF) monitoring and BIS were used throughout. Recovery of spontaneous ventilation was immediate and uneventful in all remaining treatments. A timeline summarising the clinical evolution during the first three ECT sessions is shown in Figure 1.

The patient completed all 12 planned ECT sessions without further anaesthetic complications. Anaesthetic recovery remained immediate and stable throughout the remainder of the treatment course with the rocuronium-sugammadex regimen. Her diagnosis of PD was documented in the medical record, and she was advised to communicate this information during future medical encounters. After the ECT course, she continued follow-up with psychiatry, and no further episodes of delayed recovery or adverse events were reported during clinical reassessment.

## Discussion

This case illustrates the diagnostic challenges of identifying PD during ECT, where overlapping pharmacological and procedural factors can obscure recognition of prolonged paralysis. Pseudocholinesterase, also known as butyrylcholinesterase, hydrolyses ester compounds including succinylcholine and mivacurium. Reduced enzyme activity, whether congenital or acquired, prolongs the duration of action of these agents [4,6,7]. Heterozygous individuals typically experience a 20-30% prolongation of paralysis, whereas homozygotes may remain paralysed for several hours [4-6].

In this patient, the initial prolonged emergence was attributed to extensive psychotropic and antiepileptic polypharmacy, a common feature in ECT practice that complicates the interpretation of anaesthetic responses [2,5]. The recurrence of delayed recovery during the second session prompted suspicion of PD. Although BIS monitoring indicated apparently adequate hypnosis, the peripheral nerve stimulator used for single-twitch assessment produced inconsistent responses, preventing reliable confirmation of residual block at the time.

BIS monitoring is known to be unreliable during ECT due to seizure-related EEG artefacts and postictal suppression. These factors produce abrupt fluctuations that reflect seizure dynamics rather than true hypnotic depth, and BIS should therefore not be used in isolation to judge anaesthetic adequacy in this setting [8-11]. Clinical judgement, supported by functional neuromuscular assessment, remains essential.

The patient remained mildly hypertensive and tachycardic, consistent with the sympathetic surge typically induced by the seizure [3,10]. Such haemodynamic changes may mask signs of intraoperative awareness, particularly when residual paralysis is unrecognised. The Brice Interview revealed no recall of events; however, postictal neurological suppression limits its reliability, and absence of recall does not exclude the possibility of awareness [9,11].

Once PD was confirmed by markedly reduced plasma cholinesterase activity, succinylcholine was avoided in subsequent sessions. Rocuronium followed by high-dose sugammadex provided rapid and predictable reversal independent of cholinesterase activity, an important consideration in an ECT environment outside the operating theatre. Although this strategy carries higher cost implications, it offers a high degree of safety and control in patients with known or suspected PD [4,5].

Dibucaine number testing was not available in our institution, reflecting the reality in many centres where diagnosis relies primarily on plasma enzyme activity and clinical correlation [4,5]. While additional biochemical characterisation may help distinguish genetic variants, it rarely alters acute management.

Routine neuromuscular block monitoring is strongly recommended during ECT [5,8,12]. Objective assessment helps differentiate residual paralysis from other causes of delayed awakening and enables early recognition of atypical recovery patterns, thereby enhancing patient safety.

This case underscores the importance of pharmacological vigilance, reliable neuromuscular assessment, and awareness of enzyme variability during ECT. Careful interpretation of delayed emergence is essential to prevent unrecognised paralysis and ensure safe anaesthetic practice.

## Conclusions

This case demonstrates that PD should be suspected when recovery after succinylcholine is markedly prolonged in ECT, particularly when psychotropic polypharmacy and postictal physiology make clinical assessment difficult. The reproducibility of delayed recovery across sessions and the use of quantitative neuromuscular monitoring were key to recognising the underlying cause. Substituting succinylcholine with rocuronium and reversal with sugammadex provided a reliable and predictable recovery, allowing the patient to complete the ECT course uneventfully.
